# Computational Identification and Comparative Analysis of Conserved miRNAs and Their Putative Target Genes in the *Juglans regia* and *J. microcarpa* Genomes

**DOI:** 10.3390/plants9101330

**Published:** 2020-10-09

**Authors:** Le Wang, Tingting Zhu, Karin R. Deal, Jan Dvorak, Ming-Cheng Luo

**Affiliations:** Department of Plant Sciences, University of California, Davis, CA 95616, USA; lerwang@ucdavis.edu (L.W.); tngzhu@ucdavis.edu (T.Z.); krdeal@ucdavis.edu (K.R.D.); jdvorak@ucdavis.edu (J.D.)

**Keywords:** homoeologous, miRNA, subgenome, walnut

## Abstract

MicroRNAs (miRNAs) are important factors for the post-transcriptional regulation of protein-coding genes in plants and animals. They are discovered either by sequencing small RNAs or computationally. We employed a sequence-homology-based computational approach to identify conserved miRNAs and their target genes in Persian (English) walnut, *Juglans regia,* and its North American wild relative, *J. microcarpa.* A total of 119 miRNA precursors (pre-miRNAs) were detected in the *J. regia* genome and 121 in the *J. microcarpa* genome and miRNA target genes were predicted and their functional annotations were performed in both genomes. In the *J. regia* genome, 325 different genes were targets; 87.08% were regulated by transcript cleavage and 12.92% by translation repression. In the *J. microcarpa* genome, 316 different genes were targets; 88.92% were regulated by transcript cleavage and 11.08% were regulated by translation repression. Totals of 1.3% and 2.0% of all resistance gene analogues (RGA) and 2.7% and 2.6% of all transcription factors (TFs) were regulated by miRNAs in the *J. regia* and *J. microcarpa* genomes, respectively. *Juglans* genomes evolved by a whole genome duplication (WGD) and consist of eight pairs of fractionated homoeologous chromosomes. Within each pair, the chromosome that has more genes with greater average transcription also harbors more pre-miRNAs and more target genes than its homoeologue. While only minor differences were detected in pre-miRNAs between the *J. regia* and *J. microcarpa* genomes, about one-third of the pre-miRNA loci were not conserved between homoeologous chromosome within each genome. Pre-miRNA and their corresponding target genes showed a tendency to be collocated within a subgenome.

## 1. Introduction

MicroRNAs (miRNAs) are small, 20–24 nucleotides (nt) long non-coding RNAs, which play important roles in the post-transcriptional regulation of protein-coding genes [1]. The first miRNA, *lin-4*, was discovered as a temporal regulator of larval differentiation in *Caenorhabditis elegans* [2], and thousands of additional miRNAs have been discovered since then. Their regulatory roles in various biological processes have been uncovered in many animals and plants [1,2,3,4,5,6,7,8,9]. MicroRNAs are located within intronic, exonic, and intergenic regions [10]. MicroRNA genes are transcribed by RNA polymerase II [11,12]. The resulting primary miRNA (pri-miRNA) transcripts are processed in the nucleus into single hairpin structures called precursor miRNAs (pre-miRNAs) by Drosha RNase III in animals [11] and Dicer-like RNase III (DCL1) in plants [1]. In animals, pre-miRNAs are exported into the cytoplasm, where they are further processed by Dicer RNase III [13] while pre-miRNAs in plants are entirely processed by DCL1 in the nucleus [1].

Mature plant miRNAs regulate their specific messenger RNAs (mRNAs) by two major mechanisms: transcript cleavage [14] and translation repression [15]. They have a great impact on plant biological processes, development in particular [16,17,18]. For example, plant miRNAs regulate the timing of flowering, stem cells of shoot meristems and embryogenic suspensor cells [6], leaf development [19], and are involved in hormone signaling [20]. Plant miRNAs can regulate responses to environmental [9,21] and biotic stresses [22] and can suppress R genes [23]. The majority of miRNA targets are transcription factors (TFs) [19,24]. Transcription factors activate or repress the transcription of their targets [25]. Therefore, miRNAs and TFs could form a regulatory network to regulate the gene expression during biological processes (reviewed by [26]).

Since the first miRNA discovery in *Arabidopsis* [1], miRNAs have been discovered in many plant species by either small RNA sequencing or computational prediction. The criteria for the annotation of plant miRNAs were codified and published [27,28]. A prerequisite for computational approaches is an accurate reference miRNA database [29], such as that compiled in miRBase [30,31]. A comprehensive homology-based in silico miRNA identification pipeline using known high-confidence miRNAs was developed for plants [29], and many miRNAs have been identified with this pipeline. 

The computational prediction of miRNAs is used here for the discovery and annotation of miRNAs in the genomes of *Juglans regia* and *J. microcarpa*. The former species is native to Asia and is widely grown in temperate regions of the world as an important nut tree crop. The latter species is a small wild tree native to North America. The two species diverged about 8 million years ago (MYA) [32]. Their genomes consist of *n*=16 chromosomes, are of similar sizes [33], and have had similar numbers of protein-coding genes annotated within them [32]. *Juglans* genomes originated by a whole genome duplication (WGD) of an ancestral genome with *n* = 8 [34] and consequently consist of two subgenomes. Eight pairs of fractionated homoeologous chromosomes are clearly discernible in *Juglans* genomes [32,34]. Within each homoeologous chromosome pair, one chromosome contains more genes, which are on average more expressed than those on its homoeologue [32]. The former chromosome was designated as the dominant chromosome (D) and the latter as the subdominant (S) chromosome within each homoeologous chromosome pair [32]. The eight dominant chromosomes constitute the dominant subgenome and the eight subdominant chromosomes constitute the subdominant subgenome within each genome [32].

Scaffold-level genome sequences were reported for both species [35,36,37], but were too fragmentary to allow detailed analyses of the structure and evolution of the genomes. The recently published reference-quality sequences for the *J. regia* and *J. microcarpa* genomes [32] facilitated the identification of miRNAs and their precursors, including their distribution on the chromosomes of both species. Comparative analysis of miRNAs and their miRNA families among homoeologous chromosome pairs can provide information about miRNA conservation between the genomes of *J. regia* and *J. microcarpa* and fractionation of miRNAs and target genes on dominant and subdominant chromosomes within the genomes. 

A sequence-homology-based approach is used here to identify conserved miRNAs and their target genes in the *J. regia* and *J. microcarpa* genomes. Functional annotation of the target genes is then used to identify pathways and processes that are primarily regulated by miRNAs. The distribution of miRNA and their target genes within genomes is compared in the two species with an emphasis on the fractionation of miRNAs and their target genes between the subgenomes. 

## 2. Material and Methods

### 2.1. Sequences

Datasets of high-confidence, mature plant miRNAs were downloaded from the miRbase release 21.0 [38] (873 miRNAs) [30] and release 22.0 (446 miRNAs) [31]. The two datasets were used as reference miRNA datasets to predict miRNAs. De novo assembled reference genome sequences of *J. regia* JrSerr_v1.0 and *J. microcarpa* Jm31.01_v1.0 [32] were used for the discovery and characterization of miRNAs and their target protein-coding genes. 

### 2.2. Computational Identification of miRNAs

Methodologies and tools developed by Alptekin et al. [29] were used to identify miRNAs in the *J. regia* and *J. microcarpa* genome sequence assemblies. High-confidence mature miRNA sequences were used as a query in homology searches against the genome sequences using the SUmirFind script. A maximum of two mismatches was allowed. With the SUmirFold script, 700 nt fragment including a hit of a mature miRNA were extracted. UNAFold was used to generate and evaluate the potential secondary structures of pre-miRNAs. Minimum free energy (MFE) values were calculated for each secondary structure. The secondary structure of pre-miRNAs with the lowest MFE value was retained and further filtered using SUmirPredictor, based on the characteristics of the miRNA precursors [27]. These steps produced final mature miRNAs and corresponding pre-miRNAs. SUmirLocator was used to analyze the genomic distribution of the miRNAs.

### 2.3. Prediction of miRNA Target Genes

To predict putative target genes of the discovered *J. regia* and *J. microcarpa* miRNAs, the mature miRNA sequences and mRNA sequences [32] were uploaded to the plant small RNA target prediction server psRNATarget [39]. The following default parameters under Schema V2 (2017 release) were used to identify target genes: the number of top target mRNAs for each small RNA was set to 200; the expectation was set to 5.0; the seed region was set to 2-13 nt; the maximum number of mismatches in the seed region was set to 2; the High-scoring Segment Pair (HSP) size (the complementarity scoring between small RNA and target mRNA) was set to 19; and the translation inhibition range was set to 10–11 nt [40].

### 2.4. Analysis of GO and KEGG Pathways

Homology searches of mRNA sequences against a non-redundant database (2020-01-08) from the National Center for Biotechnology Information (NCBI) were performed using BLASTX with an E-value of 1E-5. The top five hits were mapped to the Gene Ontology (GO) database using TBtools, which conducts gene annotations via ID-mapping [41]. The target genes were depicted in terms of their biological processes, cellular components, and molecular functions. Since some miRNAs target the same gene, we counted the number of target genes that were assigned with the same GO term and estimated the total frequencies of GO terms in *J. regia* and *J. microcarpa*, respectively. The targeted mRNA sequences were mapped to the Kyoto Encyclopedia of Genes and Genomes (KEGG) database to identify biological pathways in which they are involved.

## 3. Results

### 3.1. Computational Identification of miRNAs

Based on homology searches with mature plant miRNAs from miRBase release 22.0, 89 pre-miRNAs encoding 33 mature miRNAs and 95 pre-miRNAs encoding 32 mature miRNAs were predicted in *J. regia* and *J. microcarpa*, respectively. These miRNAs belonged to 23 miRNA families. Using mature plant miRNAs from miRBase release 21.0, 119 and 121 pre-miRNA loci were identified in the *J. regia* and *J. microcarpa* genomes, respectively. These pre-miRNAs could generate 39 and 40 mature miRNAs belonging to 27 and 26 miRNA families in the *J. regia* and *J. microcarpa* genomes, respectively (Table S1). To compare the high-confidence miRNA sets between miRBase release 21.0 and 22.0, we predicted miRNA in the *Arabidopsis* genome (TAIR10) [42] and compared the predicted miRNAs with the miRNA annotation in the *Arabidopsis* genome. Using miRBase release 21.0, we detected 40 mature miRNAs from 26 miRNA families, while 30 mature miRNAs belonging to 20 miRNA families were detected with miRBase release 22.0. All predicted miRNAs agreed with miRNA annotation in the *Arabidopsis* genome [42] except the miR162 on Chr5 (Table S2). Since more miRNAs were correctly detected with miRBase release 21.0, the subsequent analyses were performed with results of miRNA prediction based on miRBase release 21.0. The greater numbers of pre-miRNAs over mature miRNAs is due to the fact that different pre-miRNAs may be processed into the same mature miRNA. The length of the pre-miRNAs ranged from 100 to 209 nt with an average of 126 nt in the *J. regia* genome (Table S3) and from 89 to 209 nt with an average of 126 nt in the *J. microcarpa* genome (Table S4). The mature miRNAs were 17-22 nt in length, with a modal number of 21 nt in both genomes (Tables S3 and S4). Secondary structures of pre-miRNAs were predicted based on two criteria, Minimum Folding Energy (MFE) and Minimum Folding Energy Index (MFEI). The average MFE value of identified pre-miRNAs was -55.33 and the average MFEI was 0.97 for *J. regia* (Table S3). Average MFE and average MFEI values were very similar for the *J. microcarpa* pre-miRNAs, -54.81 and 0.96, respectively, (Table S4).

### 3.2. Identification of Target Genes

Identification of the miRNA target gene(s) is a prerequisite for the prediction of miRNA function. We predicted 577 and 604 target genes in the *J. regia* and *J. microcarpa* genomes, respectively. After eliminating duplications caused by the same gene being regulated by more than one miRNA, 325 and 316 unique target genes were predicted to be regulated by the 39 *J. regia* miRNAs and 40 *J. microcarpa* miRNAs, respectively (Tables S5 and S6). In plants, there are two regulatory mechanisms: transcript cleavage and translation repression. The central region of the complementary region between miRNA and the corresponding target gene is essential for transcript cleavage. The translation repression often happens if any mismatch occurs around the center of the complementary region. Among the 325 target genes in *J. regia*, 283 (87.08%) were regulated by transcript cleavage, while the remaining 42 (12.92%) were regulated by translation repression. In *J. microcarpa,* 281 (88.92%) target genes were regulated by transcript cleavage and 35 (11.08%) were regulated by translation repression (Tables S5 and S6). 

The annotation of R genes and TF in the *J. regia* and *J. microcarpa* genomes [32] was employed in the assessment of percentages of these genes regulated by miRNAs. A total of 942 and 903 resistance gene analogues (RGA) and 2046 and 2032 transcription factors (TFs) have previously been catalogued in the *J. regia* and *J. microcarpa* genomes, respectively [32]. We observed 12 (1.3%) and 18 (2.0%) RGA genes that were miRNA targets in *J. regia* and *J. microcarpa*, respectively, while 56 (2.7%) and 54 (2.6%) of the TF genes were miRNA targets, respectively. A larger proportion of TF genes were regulated by miRNAs than RGA genes in the *J. regia* genome (*p* = 0.024, Fisher exact test, Bonferroni corrected), but not in the *J. microcarpa* genome (*p* = 0.608, Fisher exact test, Bonferroni corrected).

### 3.3. Analysis of GO

**Figure 1:**
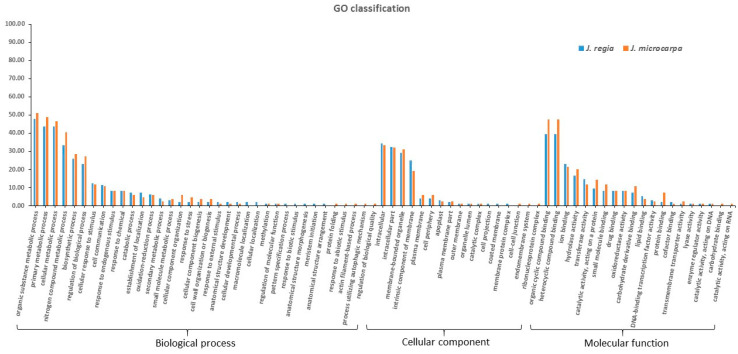
Gene Ontology (GO) functional classification of microRNA (miRNA) target genes identified in the *J. regia* and *J. microcarpa* genomes. *y*-axis represents the percentage of the number of target genes.

The *J. regia* and *J. microcarpa* target genes were functionally annotated. The GO molecular function category had target genes that were enriched in heterocyclic compound binding (GO: 1901363) and organic cyclic compound binding (GO: 0097159) in both genomes. The GO cellular component category had target genes that were mainly categorized as intracellular (GO: 0005622), intracellular part (GO: 0044424), and membrane-bounded organelle (GO: 0043227). The GO biological process category had target genes that were enriched in an organic substance metabolic process (GO: 0071704), primary metabolic process (GO: 0044238), cellular metabolic process (GO: 0044237), and a nitrogen compound metabolic process (GO: 0006807) (Figure 1; Table S7). 

### 3.4. KEGG Analysis

KEGG analysis revealed that the target genes played roles in primary metabolism and biosynthetic pathways. Of 325 *J. regia* unique targets, 104 were KEGG annotated. These protein families were involved in genetic information processing, carbohydrate metabolism, environmental information processing, and signaling and cellular processing (Table S8). Of the 316 *J. microcarpa* unique target genes, 96 were KEGG annotated. They were associated with similar protein families as those annotated in *J. regia* (Table S9). 

### 3.5. Comparative Analysis

There were no significant differences between the *J. regia* and *J. microcarpa* genomes in the numbers of pre-miRNAs (119 and 121) and miRNA families (27 and 26) (*p* = 0.94035). Within miRNA families, the numbers of different pre-miRNA loci were very similar in the *J. regia* and *J. microcarpa* genomes (*r* = 0.93, *p* < 0.0001) (Table 1). Only one family, miRNA169, was only present in the *J. regia* (Table 1) genome, where it was located on chromosomes Jr3S and Jr5D. 

**Figure 2:**
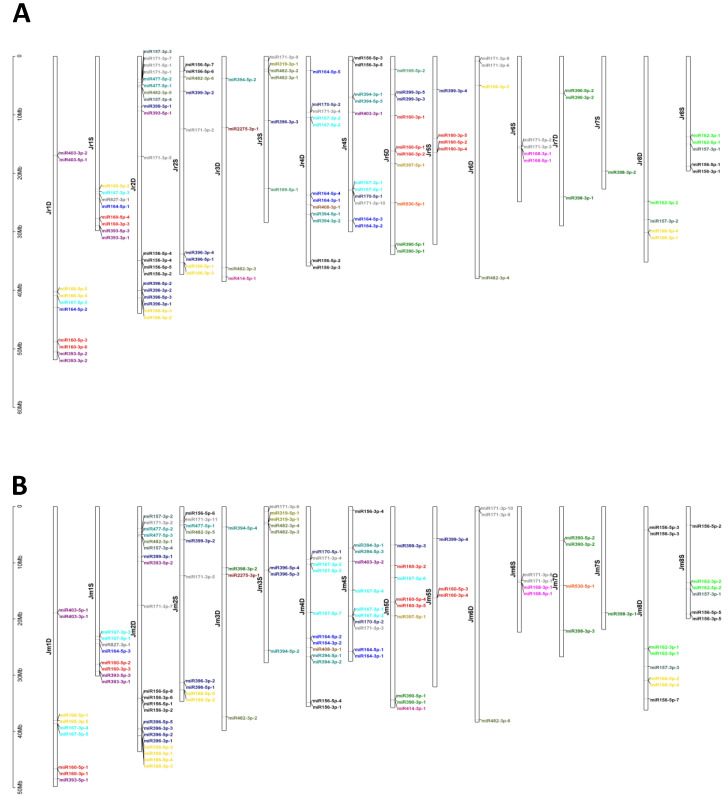
Chromosomal distribution of the pre-miRNAs in the *J. regia* (**A**) and *J. microcarpa* (**B**) genomes. The colors of the miRNA accession names indicate different miRNA families.

**Figure 3:**
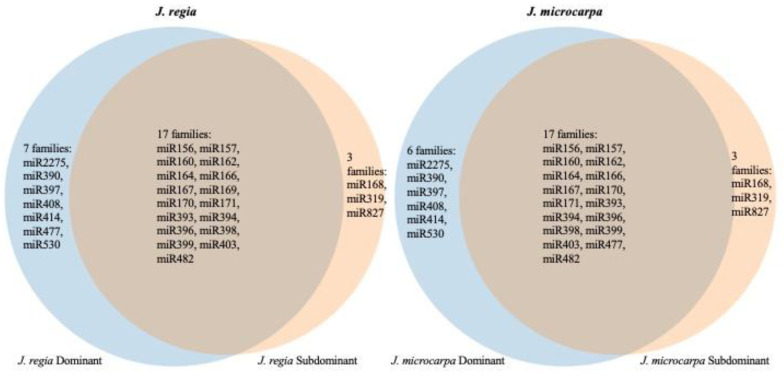
Venn diagrams of the shared and genome-specific miRNA families on dominant (blue) and subdominant (cream) chromosomes in the *J. regia* genome (**left**) and *J. microcarpa* genome (**right**).

Within the *J. regia* genome, 70 and 49 pre-miRNAs were located in the D and S subgenomes, respectively (Figure 2; Table S3). Within the *J. microcarpa* genome, 70 and 51 pre-miRNA were located in the D and S subgenomes, respectively (Figure 2; Table S4). A larger proportion of pre-miRNAs were encoded in the D subgenome than in the S subgenome if both species are considered jointly (*p* = 0.001, χ^2^ test of equal proportions on combined data, *df = 1*). Individually, *p* = 0.108 and *p* = 0.168 (χ^2^ tests, *df* = 1, corrected), for *J. regia* and *J. microcarpa,* respectively, where here and thereafter the corrected *p*-values are adjusted for two species by the Bonferroni method unless otherwise mentioned. A similar observation was made for the distribution of mature miRNAs within the two genomes. Of the 27 *J. regia* miRNA families, 24 and 20 were located in the D and S subgenomes, respectively; 17 miRNA families were in both the D and S subgenomes, 7 were specific to the D subgenome and 3 were specific to the S subgenome (Figure 3). In the *J. microcarpa* genome, of the 26 miRNA families, 23 and 20 were encoded in the D and S subgenomes, respectively; 17 families were encoded in both the D and S subgenomes, 6 families were specific to the D subgenome, and 3 families were specific to the S subgenome (Figure 3). 

A total of 577 and 604 miRNA-target gene combinations were catalogued in the *J. regia* and *J. microcarpa* genomes, and the pseudomolecule location was determined for 576 and 601 of these combinations, respectively. For the 576 *J. regia* miRNA-target gene combinations, 323 target genes were in the D subgenome and 253 were in the S subgenome. Again, a larger proportion of target genes were in the D subgenome (*p* = 0.008, χ^2^ test of equal proportions, *df* = 1, corrected). The proportion of genes that were miRNA targeted did not differ in the D and S subgenomes (*p* = 0.6798, χ^2^ test, *df* = 1, corrected). For the 601 *J. microcarpa* miRNA-target gene combinations, 370 were in the D subgenome and 231 were in the S subgenome (*p* < 0.0002, χ^2^ test of equal proportions, *df* = 1, corrected). Again, the proportion of genes that were miRNA-targeted did not differ in the D and S subgenomes (*p* = 0.180, χ^2^ test, *df* = 1, corrected). 

Target genes of miRNAs encoded in the D subgenome in *J. microcarpa* were preferentially located in the D subgenome after the expected target gene numbers were adjusted for the differences in the total numbers of genes in the two subgenomes (Table 2), as indicated by the marginally significant result. 

## 4. Discussion

Based on homology searches against the most recent release of miRBase (release 22.0), 89 and 95 pre-miRNAs were, respectively, predicted in the *J. regia* and *J. microcarpa* genomes. However, using the less recent miRBase (release 21.0), 119 and 121 pre-miRNA were predicted in the *J. regia* and *J. microcarpa* genomes, respectively. This difference mirrored the greater number of High Confidence (HC) mature plant miRNAs in the databases; there were 873 HC mature plant miRNAs in miRBase release 21.0, while there were only 446 HC mature plant miRNAs in miRBase release 22.0. Since miRBase release 21.0 appeared to be a richer resource for pre-miRNA discovery in *Juglans*, miRBase release 21.0 was employed in our analyses. To exclude the possibility that this observation is specific to the walnut genome assemblies used here, we also predicted miRNAs in the *Arabidopsis* genome (TAIR10) [42] using HC mature plant miRNAs from miRBase release 21.0 and 22.0, respectively. The predicted miRNAs from the two data sets were in line with the miRNA annotation in the *Arabidopsis* genome, except miR162 on Chr5. However, the predicted number (40 mature miRNAs) using miRBase release 21.0 was greater than the number (30 mature miRNAs) generated by release 22.0. The use of an accurate reference miRNA set is crucial for both homology and machine-learning-based miRNA mining. The HC mature plant miRNAs in miRBase release 22.1 are from eight species with 279 HC mature miRNAs. There are the fewer species and mature miRNAs among the more recent releases 22.1 (8, 279) and 22.0 (12, 446) than in 21.0 (22, 873). We therefore did not adopt the HC mature miRNAs from recent releases to detect miRNA in *J. regia* and *J. microcarpa*.

High-confidence pre-miRNAs should have MFEI values above 0.85 [43]. Our pre-miRNAs had average MFEIs of 0.97 and 0.96 for *J. regia* and *J. microcarpa,* respectively. High MFEI values of our pre-miRNAs indicate that the homology-based approach we employed identified HC pre-miRNAs. 

High-confidence pre-miRNA loci identified in our study belonged to 27 and 26 miRNA families in the *J. regia* and *J. microcarpa* genomes, respectively, and the numbers of different pre-miRNA loci within families in the two genomes were very similar. The two genomes were also similar in the distribution of pre-miRNA loci along the chromosomes and their fractionation on the dominant and subdominant homoeologues. The only exception was the miR169 family, which was only present in the *J. regia* genome. Upon further analysis, we found pre-miR169 hits in *J. microcarpa*, but those precursors were discarded from the dataset because (1) there was a mismatch in the DICER-LIKE enzyme recognition sequence of mature miRNA and (2) because more than one loop structure existed at the terminal end of the pre-miRNA and, hence, the stability of this branched loop structure was problematic [44]. These observations show that despite the divergence of the two genomes for eight MY and the evolution of *J. regia* and *J. microcarpa* on different continents, miRNAs and their precursors have been conserved. This conservation is likely a consequence of the slow evolution that characterizes genomes of woody perennials [34], but also the computation approach used for miRNA discovery. In a study similar to ours, also analyzing woody perennials, a computational method was employed in miRNA discovery in date palm and two species of oil palm [45]. The oil palm species diverged 51 MYA and both diverged 65 MYA from date palm [46]. Of 69 non-redundant miRNAs identified among the three species, 45 miRNAs were shared by all of three species; only one and six miRNAs were unique to an oil palm species and 10 were unique to date palm. Thus, as in our study, the computational approach preferentially identified conserved miRNAs.

In both genomes, the most abundant pre-miRNAs were in families miR156, miR160, miR166, and miR171. MicroRNAs miR156 and miR166 are conserved across the plant kingdom and play important roles in flower development among flowering plants [47]. In addition to these families, miR396 was found to be conserved in all vascular plants [48] and also was represented by a large number of pre-miRNAs in both genomes. Although miR397 and miR398 have been shown to be present in all seed plants [48], and miR403 has been shown to be conserved among angiosperms [48], these families consisted of only one to three pre-miRNAs in both genomes. Thus, the antiquity of an miRNA family may not have led to its diversification during *Juglans* evolution. 

The availability of reference-quality genome sequences for *J. regia* and *J. microcarpa* allowed us to analyze the distribution of pre-miRNA loci within genomes. Plant pre-miRNAs are mostly scattered across the genome, with only a minority of them forming clusters [49]. This pattern contrasts with the distribution of animal pre-miRNAs, which are often clustered. The distribution of pre-miRNAs in both *Juglans* genomes is consistent with the pattern described for other plants, as the detected pre-miRNAs were scattered in both genomes. The closest distance between miRNAs, miR157 and miR482, was 14,527 bp on chromosome 2D in *J. regia*, and 19,574 bp on chromosome 2D in *J. microcarpa*.

The *J. regia* and *J. microcarpa* genomes differed in only one miRNA family, which included two pre-miRNA loci, while there were substantial differences in the pre-miRNA loci between subgenomes within the genomes (Figure 3). Homoeologous chromosomes within the *J. regia* and *J. microcarpa* genomes shared only 65 and 67% of the pre-miRNA loci, respectively. The rest of them were rendered unique by the fractionation of the D and S homoeologous chromosomes. The minor divergence between *J. regia* and *J. microcarpa* genomes, but substantial divergence within the genomes, is consistent with the estimated age of the *J. regia* and *J. microcarpa* genome divergence, 8 MYA, and the age of the WGD, which took place near the Cretaceous-Paleogene boundary, about 65 MYA [34].

We identified 325 and 316 unique target genes for *J. regia* and *J. microcarpa* miRNAs, respectively, which facilitated functional analyses of *Juglans* miRNAs. No difference in the function of target genes were observed between *J. regia* and *J. microcarpa* according to the GO and KEGG annotation of target genes. These results showed that the functions of the miRNAs identified are also conserved in both species. The distribution of target genes within the *J. regia* and *J. microcarpa* genomes, as well as the distribution of pre-miRNAs, mirrors the distribution of protein-coding genes. More pre-miRNAs are encoded in the D subgenome than in the S subgenome. Thus, the same evolutionary process that produced asymmetric fractionation of protein-coding genes [32] also produced asymmetric fractionation of the pre-miRNAs and their target genes. We suggest that the asymmetric fractionation of genes on homoeologous chromosomes within *Juglans* genomes is due to weaker purifying selection acting on deletions of genes in the S subgenome than in the D subgenome, paralleling the levels of transcription [32]. We reason that a deletion of a pre-miRNA locus or a target gene will have a weaker phenotypic effect if the gene is weakly expressed than if it is strongly expressed. 

Disease-resistance genes (R genes) have been identified in *J. regia* and *J. microcarpa*. Although *J. microcarpa* is important in the development of disease-resistant rootstocks for walnut breeding, there are only minor differences in the number of R genes between *J. regia* (942) and *J. microcarpa* (903) [32]. We found more R genes are miRNA target genes in *J. microcarpa* than in *J. regia*. In the wild *J. microcarpa*, more R genes are miRNA-regulated than in the cultivated *J. regia*, not just that there are more R genes. Transcription factors are key regulators of gene expression at transcriptional and post-transcriptional levels (reviewed [26]), and are themselves regulated by miRNA [50]. There were no differences in TFs between the two *Juglans* species [32] and similar TFs are regulated by miRNA in both. 

Lastly, miRNA precursors and its target gene show collocated to the same subgenome? This observation was marginally true in the D subgenome of *J. microcarpa* but nowhere else, suggesting that miRNA precursor and its target gene collocation has been of little, if any, biological significance during *Juglans* genome evolution. 

## Figures and Tables

**Table 1:** MicroRNA families identified in *J. regia* and *J. microcarpa*.

	*J. regia*	*J. microcarpa*
miRNA family	Number of pre-miRNAs	Number of pre-miRNAs
miR156	12	14
miR157	4	4
miR160	11	9
miR162	3	4
miR164	7	5
miR166	10	10
miR167	6	11
miR168	2	2
miR169	2	0
miR170	2	2
miR171	12	11
miR2275	1	1
miR319	1	2
miR390	4	4
miR393	5	4
miR394	5	6
miR396	7	8
miR397	1	1
miR398	2	3
miR399	5	4
miR403	3	3
miR408	1	1
miR414	1	1
miR477	4	3
miR482	6	6
miR530	1	1
miR827	1	1
Total	119	121

**Table 2:** Observed numbers of target genes by miRNAs located in the D and S subgenomes in *J. regia* and *J. microcarpa* genomes and expected numbers based on the total numbers of genes in the D and S subgenomes.

Genome	Subgenome	Genes (No.)	Observed and (Expected) Target Genes Regulated by miRNA Located in D Subgenome (No.)	Observed and (Expected) Target Genes Regulated by miRNA Located in S Subgenome (No.)
*J. regia*	D	18,179	202 (201.0) ^†^	121 (133.6)
	S	13,107	144 (145.0)	109 (96.4)
			*p* = 1.00 **	*p* = 0.368
*J. microcarpa*	D	17,093	226 (205.2)	144 (144.2)
	S	12,304	127 (147.8)	104 (103.8)
			*p* = 0.092	*p* = 1.00

^†^ Expected numbers of target genes computed from the numbers of genes in the D and S subgenomes. ** *p*-values were obtained from χ^2^ tests with *df* = 1 and Bonferroni corrections for four tests.

## Supplementary Materials

The following are available online at https://www.mdpi.com/2223-7747/9/10/1330/s1: Table S1 Locations of miRNA families and the numbers of pre-miRNA loci on the *J. regia* (Jr) and *J. microcarpa* (Jm) chromosomes, Table S2 Comparisons between miRNA prediction in TAIR10 and miRNA annotation in TAIR10, Table S3 Characteristics of mature miRNAs and pre-miRNAs identified in *J. regia*, Table S4 Characteristics of mature miRNAs and pre-miRNAs identified in *J. microcarpa*, Table S5 Chromosome locations of target genes and miRNAs in the *J. regia* genome and their regulation by miRNAs, Table S6 Chromosome locations of target genes and miRNAs in the *J. microcarpa* genome and their regulation by miRNAs, Table S7 Functional classification for targets genes in the *J. regia* and *J. microcarpa* genomes, Table S8 List of KEGG for the miRNA targets identified in *J. regia*, Table S9 List of KEGG for the miRNA targets identified in *J. microcarpa*.
